# Predictive value of early amplitude integrated electroencephalogram (aEEG) in sleep related problems in children with perinatal hypoxic-ischemia (HIE)

**DOI:** 10.1186/s12887-021-02796-9

**Published:** 2021-09-18

**Authors:** Qiuyan Tian, Yizhi Pan, Zheng Zhang, Mei Li, Li-xiao Xu, Min Gong, Po Miao, Xiaolu Jiang, Xiaofeng Yang, Chen-Xi Feng, Jian Pan, Yun Yu, Bin Sun, Xin Ding

**Affiliations:** 1 Soochow Key Laboratory of Prevention and Treatment of Child Brain Injury, Children’s Hospital of Soochow University, No.92 Zhongnanjie Road, Suzhou, 215025 P.R. China; 2 Pediatrics Research Institute, Children’s Hospital of Soochow University, Suzhou, 215025 P.R. China; 3 Department of Neonatology, Children’s Hospital of Soochow University, Suzhou, 215025 P.R. China

**Keywords:** Hypoxic-Ischemic encephalopathy (HIE), Sleep problems, aEEG, Circadian rhythmic issues, Correlation

## Abstract

**Background:**

While great attention has been paid to motor and cognitive impairments in children with neonatal Hypoxic-Ischemic Encephalopathy (HIE), sleep related circadian rhythm problems, although commonly present, are often neglected. Subsequently, no early clinical indicators have been reported to correlate with sleep-related circadian dysfunction during development.

**Methods:**

In this study, we first analyzed patterns of the amplitude integrated electroencephalogram (aEEG) in a cohort of newborns with various degrees of HIE. Next, during follow-ups, we collected information of sleep and circadian related problems in these patients and performed correlation analysis between aEEG parameters and different sleep/circadian disorders.

**Results:**

A total of 101 neonates were included. Our results demonstrated that abnormal aEEG background pattern is significantly correlated with circadian rhythmic (*r* = 0.289, *P* = 0.01) and breathing issues during sleep (*r* = 0.237, *P* = 0.037). In contrast, the establishment of sleep–wake cycle (SWC) showed no correlation with sleep/circadian problems. Detailed analysis showed that summation of aEEG score, along with low base voltage (*r* = 0.272, *P* = 0.017 and *r* = -0.228, *P* = 0.048, respectively), correlates with sleep circadian problems. In contrast, background pattern (BP) score highly correlates with sleep breathing problem (*r* = 0.319, *P* = 0.004).

**Conclusion:**

Abnormal neonatal aEEG pattern is correlated with circadian related sleep problems. Our study thus provides novel insights into predictive values of aEEG in sleep-related circadian problems in children with HIE.

**Supplementary Information:**

The online version contains supplementary material available at 10.1186/s12887-021-02796-9.

## Introduction

Neonatal hypoxic-ischemic encephalopathy (HIE), dependent on its severity, can lead to a broad spectrum of chronic neurological deficits [1–4]. While extensive clinical studies focused on diagnosing and improving motor and cognitive impairments [4–8], little attention is paid to other clinical aspects of patients with neonatal HIE.

Accumulating evidence showed that neonatal HIE patients often show a delayed onset of the sleep–wake cycle (SWC) and, subsequently, develop sleep disorders [9–11]. In light of this, our recent findings demonstrate that sleep-related circadian rhythmic issues are highly associated with children of moderate or mild neonatal HIE [12]. Although chronic sleep problems significantly compromise children’s and their caregivers’ life quality, these issues, unfortunately, are often put aside by clinicians [13]. Consequently, no perinatal management and clinical prognostic indicators have been reported for sleep-related circadian disorders in children with neonatal HIE.

Different from tradition electroencephalogram (EEG), amplitude integrated electroencephalogram (aEEG) achieves sustainable monitoring of brain electrocortical activity. In current neonatology, aEEG is widely used, along with other imaging tools, for outcome prediction in neonates with HIE [14–23]. An exclusive application of aEEG is to monitor the onset and quality of SWC. Although previous studies showed that aEEG serves as a good predictive tool for neurological development [9, 11, 24, 25], whether and how aEEG patterns correlate with sleep-related circadian disorders remain largely unknown.

To investigate whether aEEG patterns show prognostic value in developmental circadian disorders, we analyzed aEEG patterns in a cohort of newborns with various degrees of HIE. In follow-up telephone interviews, we collected information relating to sleep and circadian rhythm problems and performed correlation analysis between aEEG patterns and different sleep/circadian disorders. Our study thus sheds new light into the predictive value of aEEG in neonates with HIE for circadian rhythm disorders.

## Material and methods

### Participants

Our research protocol was approved by IRB of Children’s Hospital of Soochow University (IRB approval number: 2017038). All participants received consent form their parents. We initially included 101 neonates who were born in local hospital, diagnosed for HIE (1 min Apgar score < 7), and transferred to Children’s Hospital of Soochow University within 1wk after birth between Jan. 2016 and Jan. 2018. Within the 73 cases that were diagnosed as severe HIE, 16 of them were transferred to our hospital late than 6 h after birth and thus were ineligible for hypothermia treatment [26–29]. The left were eligible for hypothermia treatment. However, due to lack of devices and personnel, we did not implement hypothermia treatment for neonates prior to Jan. 2019. Follow-up interviews of neonates with severe HIE revealed that 2 died < 2 yr, 5 with cerebral palsy, 2 with secondary epilepsy, 8 with language developmental disorders and 28 with various degrees of developmental retardation. Exclusion criteria include: gestational age < 37 or > 42 weeks, major ischemic stroke, congenital malformations, chromosomal abnormalities and central nervous infections. General information of participants was listed in Table 1.

**Table 1:** Characteristics of the participants with neonatal HIE

aEEG BP	Normal	Abnormal	*t/χ*^*2*^	*P*
Charac-teristics				
Gestational age (d)	286.93 ± 50.94	275.43 ± 7.17	t = 1.534	0.128
Birth weight (g)	3360.46 ± 516.49	3311.70 ± 519.93	t = 0.472	0.638
Sex: Male	35	33		
Female	19	14	χ^2^ = 0.333	0.564
Delivery method				
Natural birth	32	30		
Caesarean section	22	17	χ^2^ = 0.221	0.638
HIE degree				
Mild to moderate	15	9		
Severe	39	38	χ^2^ = 0.603	0.438

Note: for gestational age and birth weight, data are present as mean ± s.d

### aEEG recording and pattern analysis

We placed a pair of biparietal electrodes at P3/P4 for aEEG recording (Natus 580-NLICU1, USA). The aEEG recording was continuously performed for 24 h, immediately after transferring to our hospital, with a paper speed of 6 cm/h. The initial time for aEEG recording was within 72 h after birth (35.128 ± 19.953 h).

**Fig. 1:**
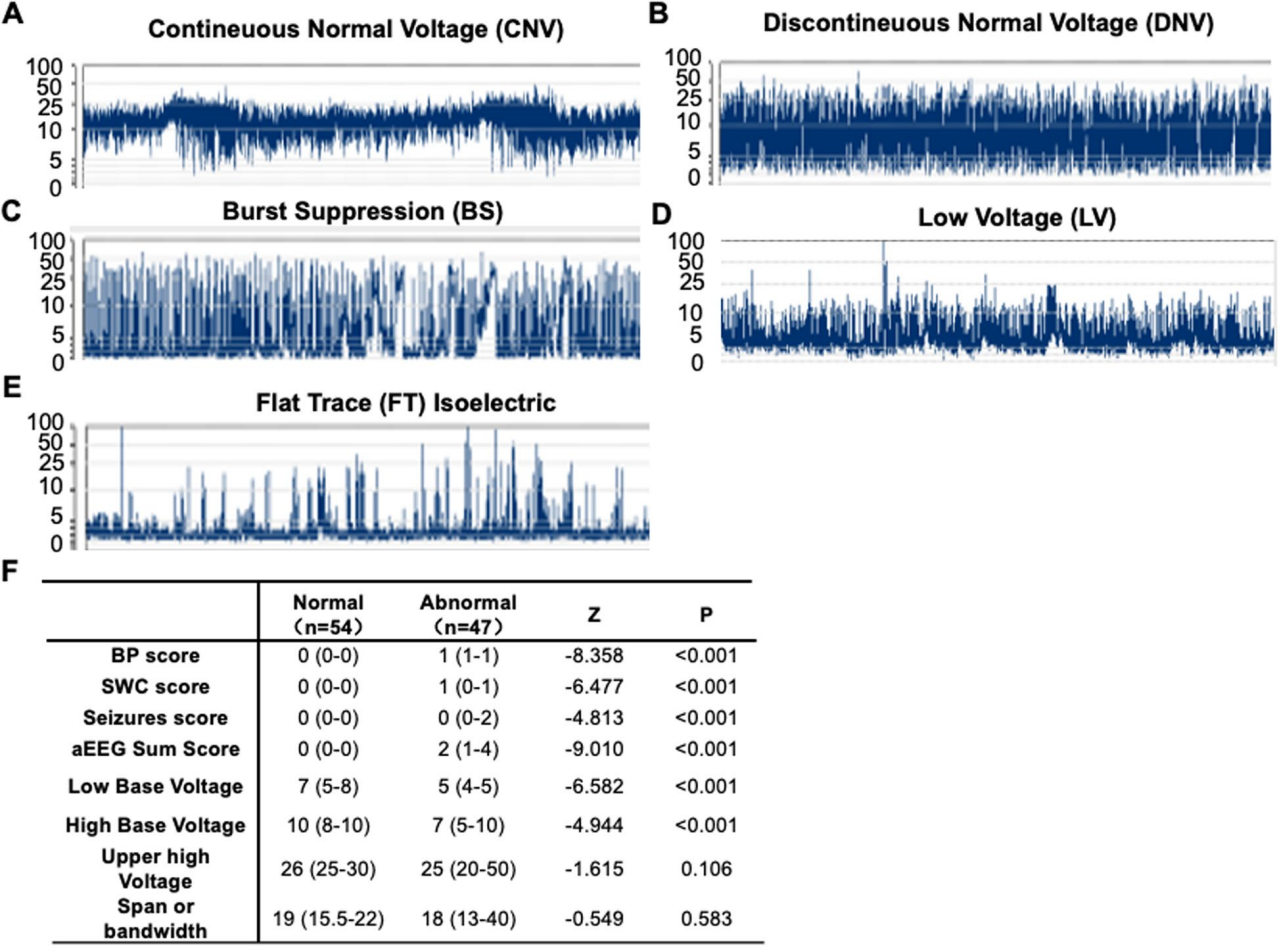
aEEG background patterns.** (A-E)** Representative traces showing different pattern of aEEG recordings in patients with neonatal HIE. **(F)** Table showing characteristic parameters in neonatal patients with normal or abnormal aEEG BP. Data are presented as median (interquartile), with Mann–Whitney U test applied

The aEEG patterns were analyzed by 2 independent technicians. The following parameters, according to [14] and others [11, 30],were used for pattern analysis:

Background pattern (BP) score: a 0–2 scale reflecting its abnormality degree was used to calculate BP: 0 for continuous normal voltage (CNV), 1 for discontinuous normal voltage (DNV), and 2 for the presence of burst suppression (BS) /continuous low voltage (LV) /flat trace (FT). The representative traces of CNV, DNV, BS, LV and FT were present in Fig. 1. During each 6-h period, we calculated an integrated score as: BP score = [(% CNV × 0) + (% DNV × 1) + (% BS/LV/FT × 2)]/100.
SWC score: a 0–2 scale was used to calculate SWC: 0 for mature, 1 for immature, and 2 for no. During each 6-h period, we calculated an integrated score as: SWC score = [(% mature × 0) + (% immature × 1) + (% no × 2)]/100.
Seizures score: a 0–2 scale was used to calculate seizures score: 0 for no, 1 for single, and 2 for repetitive seizures/status epilepticus. During each 6-h period, we calculated an integrated score as: Seizures score = [(% no × 0) + (% single × 1) + (% repetitive seizures/status epilepticus × 2)]/100.
4.aEEG sum score: aEEG summation score by adding 1–3 (ranging from 0–6).
Low base voltage (mV): the lower edge of the tracing.
High base voltage (mV): the lower margin during high activity.
Upper high voltage (mV): the upper edge of the tracing.
Span or bandwidth (mV): the difference between 6 and 7.

### Investigation of sleep and circadian rhythm problems

We investigated sleep and circadian rhythm problems by telephone interview in the cohort of HIE patients who received aEEG recordings during their hospitalization (see above section) using a self-designed questionnaire that was used in a previous study [12]. Telephone interview was completed when children were between 26 to 42 months. In brief, we include 7 variables: 1) Unfixed nocturnal sleep-onset time; 2) Extensive settling time (> 20 min); 3) Insufficient sleep (< 11 h); 4) Change in daily sleep schedule; 5) Snore loudly; 6) Sleep breathing problems; 7) Frequent wakes at night (> 2 times/night). Note: detailed description for each variable please refer to [12]. For individual variables, we applied a 0–2 scale: 0 for never happened in the past 6 months, 1 for 3 times/week in the past 6 months, and 2 for > 3 times/week in the past 6 months. The sum of all variables is attributed as sleep score. For each variable, we averaged results from two interviews with a 3-week interval. Exclusion criteria: 1) Refuse of parents or guardian to provide detailed information during telephone interview; 2) Taking sleep affective medications (e.g. anticonvulsant, sedatives, and neurostimulant drugs) within the past months before telephone interview; 3) A follow-up physical examination that discovered traumatic injuries and/or developmental abnormalities leading to severe up-airway obstruction, if sleep breathing problem was identified during telephone interview.

### Statistical analysis

Quantitative variables were compared by *t*-test, and qualitative variables were compared by the χ^2^ test or Fisher’s exact test. Skew distribution data was presented as median (interquartile) and the differences between groups were examined by nonparametric test. The correlation between aEEG/SWC and daily sleep parameters, and aEEG parameters and daily sleep parameters were described using spearman correlation coefficient, with P values present. The Statistical analyses were conducted using IBM SPSS Statistics version 25.0 software. A *P* value less than 0.05 (two-sided) indicated statistical significance.

## Results

### General patient characterization and aEEG background pattern

We included 101 neonates with HIE that were transferred to our hospital within hours after birth. The mean gestational age was 281.57 ± 37.84 d, and the mean birth weight was 3337.77 ± 516.08 g, with 68 males and 33 females. aEEG recording was started at a median age of 36 h (35.13 ± 19.96) for 24 h. Within them, 53.5% (54/101) showed normal background pattern (BP) with continuous normal voltage (CNV) recordings (Fig. 1A). The BP of the left (47/101) showed abnormalities including discontinuous normal voltage (DNV), burst suppression (BS), low voltage (LV) and flat trace (FT) (Fig. 1B-E). To investigate the predictive roles of aEEG patterns in neonates with HIE, we then divided the cohort in two groups: normal or abnormal aEEG BP and discovered no significant differences in gestational age, weight, sex, and the degree of HIE (Table 1). In addition, the degrees of HIE were independent of these characteristics (additional file 1). To perform detailed analysis of aEEG recordings, we scored aEEG recordings based on the frequency of normal vs abnormal BPs, the establishment of SWC, and the occurrence of seizures and quantified parameters that reflect brain activity and the variability of recorded signal (see method section for aEEG recording). Among these parameters, aEEG BP, SWC, seizures scores and their sum, along with low and high base voltages, indicators for cerebral activity, are statistically different between normal and abnormal BP groups (Fig. 1F). In contrast, the upper high voltage and bandwidth are independent from aEEG normality and thus could serve as reference parameters (Fig. 1F).

### Neonates with abnormal aEEG BP tend to develop specific sleep problems

In a serial of follow-ups, we investigated the sleep quality and maintenance of normal behavioral rhythm activities in HIE neonates who received aEEG recordings. Based on the results of questionnaire, while the total sleep scores showed no statistical differences, neonates with abnormal aEEG BP tend to have more problems in maintaining daily sleep schedule, frequent night wake up and breathing during sleep (Table 2). In contrast, such differences were not observed in neonates with various degrees of HIE (additional file 2). These results indicated that aEEG BP normality might correlate with sleep-related circadian rhythm and breathing problems. To further test this, we calculated the correlations between seven sleep-related parameters with aEEG BP normality. In light with the comparison between neonates with either normal or abnormal aEEG BP groups, we found that aEEG BP is correlated with sleep schedule change and breathing problems (Table 3). Consistent with previous findings, the establishment of SWC was severely compromised in neonates with severe HIE (SWC score: 0.10 ± 0.36 (mild and moderate HIE) vs 0.66 ± 0.80 (severe HIE), *t* test, P < 0.001). However, the early establishment of SWC is not correlated with any developmental sleep-related issues (Table 3).

**Table 2:** Characterization of sleep problems in neonatal HIE patients with normal or abnormal aEEG patterns

Sleep problems	Abnormal aEEG BP (n = 41)			Normal aEEG BP (n = 39)			*P*
	0	1	2	0	1	2	
Unfixed sleep on-set time	28	4	9	30	5	4	0.305
Settling time (> 20 min)	23	11	7	24	9	10	0.510
Insufficient sleep (< 11 h)	24	17	0	20	6	9	0.562
Changes in daily sleep schedule	16	9	16	24	7	8	0.036
Snore loudly	30	11	0	32	6	1	0.384
Sleep breathing problems	34	7	0	38	1	0	0.032
Frequent night wake-up	24	16	1	19	6	14	0.046
Sleep score	3.44 $\pm$ 1.75			3.38 $\pm 2$.57			0.582

**Table 3:** Correlations between sleep problems and normal aEEG pattern or establishment of SWC

		aEEG BP	SWC
Unfixed sleep	r	0.102	-0.022
on-set time	*P*	0.375	0.847
Settling time	r	-0.068	0.041
(> 20 min)	*P*	0.554	0.719
Insufficient sleep	r	-0.064	-0.165
(< 11 h)	*P*	0.577	0.149
Changes in daily	r	0.289	0.137
Sleep schedule	*P*	0.010	0.232
Snore loudly	r	0.119	-0.005
	*P*	0.298	0.964
Sleep breathing	r	0.237	-0.033
problems	*P*	0.037	0.772
Frequent	r	-0.095	-0.025
night wake-up	*P*	0.408	0.825
Sleep score	r	0.133	0.020
	*P*	0.245	0.861

### aEEG parameters correlate with distinct sleep related problems

To investigate whether and how aEEG recording might play predictive roles in sleep related problems, we then performed correlation studies between individual aEEG parameters and sleep-related variables. Within these parameters, we identified that aEEG sum score and low base voltage are correlated with changes of daily sleep schedules, a circadian rhythm related issue. In contrast, BP score is correlated with sleep breathing problems (Table 4). These results indicated that individual aEEG parameters show distinct predictive roles in sleep related problems during child development.

**Table 4:** Correlations between aEEG parameters and sleep problems in patients with neonatal HIE

Sleep problems		Unfixed sleep on-set time	Settling time	Insufficient Sleep (< 11 h)	Changes in daily sleep schedule	Snore loudly	Sleep breathing problems	Frequent night wake-up	Sleep score
aEEG parameters									
BP score	r	0.042	-0.024	-0.074	0.208	0.199	0.319	-0.006	0.150
	*P*	0.712	0.833	0.521	0.068	0.081	0.004	0.956	0.189
SWC score	r	-0.035	0.011	-0.179	0.137	0.024	-0.022	-0.042	0.000
	*P*	0.759	0.920	0.116	0.233	0.837	0.846	0.715	0.998
Seizures score	r	-0.007	0.003	-0.197	0.078	0.070	0.051	-0.048	-0.029
	P	0.949	0.978	0.084	0.500	0.541	0.660	0.679	0.802
aEEG sum	r	0.030	-0.020	-0.173	0.272	0.093	0.181	-0.038	0.095
	*P*	0.799	0.864	0.132	0.017	0.420	0.116	0.746	0.411
Low base	r	-0.020	0.087	0.121	-0.228	0.216	-0.135	0.028	-0.106
voltage	*P*	0.866	0.454	0.300	0.048	0.061	0.244	0.812	0.363
High base	r	0.053	0.037	0.045	-0.105	0.200	-0.158	-0.015	-0.070
voltage	*P*	0.652	0.750	0.697	0.367	0.084	0.172	0.900	0.546
Upper high	r	0.031	-0.016	-0.069	-0.141	0.093	-0.035	-0.155	-0.169
voltage	*P*	0.789	0.890	0.551	0.223	0.422	0.767	0.182	0.145
Span or	r	0.046	0.000	-0.073	-0.105	0.038	0.032	-0.157	-0.121
bandwidth	*P*	0.695	0.999	0.532	0.369	0.744	0.787	0.177	0.298

## Discussion

Compared to other neurological outcomes, sleep-related circadian issues in neonates with HIE are paid much less attention. This study sought to investigate the correlations between recordings from aEEG and sleep-related problems during development. By performing follow-up interviews in neonatal HIE with early aEEG records, we collected data of their sleep quality and circadian rhythm related problems. Our results revealed that normal aEEG BP, but not the establishment of SWC, is correlated with certain sleep-related problems. Further analysis indicated that individual aEEG parameters might have distinct predictive value in sleep-related problems. Our study thus shed novel insights into the use of aEEG data, a commonly used bed-side tool to monitor brain activities in neonates with HIE, as a prognostic tool in predicting sleep-related issues during development.

Previous studies showed that aEEG is a reliable tool to monitor the establishment of SWC, the onset of which serves a good indicator for effectiveness of hypothermia treatment and neurological outcome [9, 11]. In addition, aEEG itself, often along with other parameters, showed good predictive value for long-term prognosis [14, 16]. In certain cases, using aEEG might serves better than the onset of SWC. For example, [31] showed that time to normal aEEG is a better predictor than time to SWC in HIE neonates treated with hypothermia. Although the recorded time might significantly affect its predictive roles [20, 32]. Nevertheless, these studies indicated that predictive values of aEEG might across a broad spectrum in neonates with HIE. In light of this, our findings showed that aEEG BP, but not SWC, correlates with specific sleep-related problems, probably due to its higher sensitivity to reflect effects of HIE on the establishment of early circadian rhythm.

Two aEEG parameters: 1) the summative aEEG score reflecting combined abnormality in aEEG BP, establishment of SWC and seizure occurrence and 2) the low base voltage reflecting minimal cerebral activity during quiet sleep [33] show concrete correlation with sleep-related circadian rhythm problem. These results established the relationship between initial establishment of sleep in neonates and later circadian rhythm control in children. On the other hand, the abnormal BP pattern itself might indicate widespread cortical lesions that will damage corticofugal connections responsible for precise coordinating of muscles in the upper airway and thus cause breathing problems during sleep. In our future studies, we will have a chance to test the predictive value of aEEG parameters in sleep related problems in children with neonatal HIE. There are certain limitations of this study. First, the sample size is relatively small and, as a retrospective study, some patients were not included due to lack of response in follow-ups. Second, there are certain imperfect links in the method. For instance, video monitoring, which is commonly used to detect suspicious seizures, apneas and monitor special events such as breastfeeding, examination and treatment was not coupled with aEEG recording [34–36]. In addition, it will be interesting to compare circadian rhythm outcomes between neonates with or without hypothermia treatment in future. Nevertheless, our study provides potential prognostic values of aEEG and helps preventative adjustment of circadian rhythm disorders in children with neonatal HIE.

## Supplementary Information

**Additional file 1.** Characteristics of the participants with neonatal HIE.

**Additional file 2.** Characterization of sleep problems between mild/moderate and severe HIE patients.

## Data Availability

The datasets generated during and/or analyzed during the current study are available from the corresponding author on reasonable request.

## Abbreviations

aEEG: Amplitude integrated electroencephalogram
HIE: Hypoxic-ischemia
SWC: Sleep–wake cycle
BP: Background pattern
