# Supplementary material for: Predictive value of early amplitude integrated electroencephalogram (aEEG) in sleep related problems in children with perinatal hypoxic-ischemia (HIE)
Source: BMC Pediatr. 2021 Sep 18;21:410. doi: 10.1186/s12887-021-02796-9 (PMC8449491; doi:10.1186/s12887-021-02796-9)
Supplement: Supplementary file 1 — Additional file 1. Characteristics of the participants with neonatal HIE. [file 12887_2021_2796_MOESM1_ESM.docx]

| Normal aEEG BP  Charac-  teristics | Mild/Middle | Severe | t/χ^2^ | P |
| --- | --- | --- | --- | --- |
| Gestational  age (d) | 277.90 ± 8.25 | 275.31 ± 7.41 | t=1.011 | 0.317 |
| Birth weight (g) | 3387.32 ± 534.36 | 3275.77 ± 464.94 | t=0.675 | 0.503 |
| Gender |  |  |  |  |
| Male | 25 | 9 |  |  |
| Female | 16 | 4 | χ^2^=0.288 | 0.591 |
| Delivery method |  |  |  |  |
| Natural birth | 27 | 3 |  |  |
| Caesarean section | 14 | 10 | χ^2^=7.315 | 0.007 |

**Additional File 1**

**Characteristics of the participants with neonatal HIE**

| Abnormal aEEG BP  Charac-  teristics | Mild/Middle | Severe | t/χ^2^ | P |
| --- | --- | --- | --- | --- |
| Gestational  age (d) | 275.40 ± 6.75 | 275.43 ± 7.37 | t=-0.013 | 0.990 |
| Birth weight (g) | 3302.00 ±650.42 | 3314.32 ±489.52 | t=-0.066 | 0.948 |
| Gender |  |  |  |  |
| Male | 8 | 25 |  |  |
| Female | 2 | 12 | χ^2^=0.582 | 0.446 |
| Delivery method |  |  |  |  |
| Natural birth | 6 | 21 |  |  |
| Caesarean section | 4 | 16 | χ^2^=0.034 | 0.854 |
