# Supplementary material for: Predictive value of early amplitude integrated electroencephalogram (aEEG) in sleep related problems in children with perinatal hypoxic-ischemia (HIE)
Source: BMC Pediatr. 2021 Sep 18;21:410. doi: 10.1186/s12887-021-02796-9 (PMC8449491; doi:10.1186/s12887-021-02796-9)
Supplement: Supplementary file 2 — Additional file 2. Characterization of sleep problems between mild/moderate and severe HIE patients. [file 12887_2021_2796_MOESM2_ESM.docx]

**Additional File 2**

**Characterization of sleep problems between mild/moderate and severe HIE patients**

|  | Mild/Moderate | Severe | *t* | *P* |
| --- | --- | --- | --- | --- |
| Unfixed sleep on-set time | 0.53±0.81 | 0.40±0.74 | 0.717 | 0.476 |
| Settling time (> 20 min) | 0.72±0.85 | 0.60±0.74 | 0.669 | 0.506 |
| Insufficient sleep (< 11h) | 0.61±0.80 | 0.45±0.60 | 0.999 | 0.321 |
| Changes in daily sleep schedule | 0.75±0.87 | 0.83±0.90 | -0.367 | 0.715 |
| Snore loudly | 0.19±0.47 | 0.25±0.45 | -0.535 | 0.594 |
| Sleep breathing problems | 0.06±0.23 | 0.15±0.36 | -1.338 | 0.185 |
| Frequent night wake-up | 0.56±0.74 | 0.53±0.68 | 0.188 | 0.851 |
| Sleep score | 3.42±2.67 | 3.20±1.67 | 0.429 | 0.669 |
| BP scores | 0.14±0.35 | 0.76±0.69 | -5.766 | <0.001 |
| SWC Scores | 0.10±0.36 | 0.66±0.80 | -4.574 | <0.001 |
| Seizures scores | 0.04±0.28 | 0.62±0.90 | -4.392 | <0.001 |
| AEEG Sum | 0.27±0.70 | 2.08±2.05 | -5.951 | <0.001 |
| Low base voltage | 6.31±1.50 | 4.98±1.60 | 4.323 | <0.001 |
| High Base voltage | 8.76±1.56 | 7.52±2.52 | 2.995 | 0.003 |
| Upper high voltage | 28.51±7.45 | 35.56±24.15 | -1.990 | 0.049 |
| Span or band width | 19.73±7.20 | 28.02±24.44 | -2.323 | 0.022 |
